# Gendered race: are infants’ face preferences guided by intersectionality of sex and race?

**DOI:** 10.3389/fpsyg.2015.01330

**Published:** 2015-09-03

**Authors:** Hojin I. Kim, Kerri L. Johnson, Scott P. Johnson

**Affiliations:** Department of Psychology, UCLA Baby Lab, University of California, Los Angeles, Los Angeles, CA, USA

**Keywords:** sex and race categorization, infant face preference, social development, social cognitive development, sex differences

## Abstract

People occupy multiple social categories simultaneously (e.g., a White female), and this complex intersectionality affects fundamental aspects of social perception. Here, we examined the possibility that infant face processing may be susceptible to effects of intersectionality of sex and race. Three- and 10-month-old infants were shown a series of computer-generated face pairs (5 s each) that differed according to sex (Female or Male) or race (Asian, Black, or White). All possible combinations of face pairs were tested, and preferences were recorded with an eye tracker. Infants showed preferences for more feminine faces only when they were White, but we found no evidence that White or Asian faces were preferred even though they are relatively more feminized. These findings challenge the notions that infants’ social categories are processed independently of one another and that infants’ preferences for sex or race can be explained from mere exposure.

## Introduction

Studies of infant face preference represent an important opportunity to inform theories of social cognitive development, in particular the means by which infants determine critical features of social categories (Ramsey et al., 2005) and the means by which social context influences recognition of individuals from specific groups (Scott et al., 2007). Moreover, understanding infant face preferences may help reveal the developmental origins of stereotypes and prejudice (given that preverbal infants lack direct knowledge of group characteristics), if these origins are at least partly perceptual in nature. Contemporary theories of face processing appeal to results from studies of prototype formation, prototype preference, and intermodal matching in providing important evidence for an asymmetry in the development of infants’ categorical knowledge of female and male faces, such that knowledge of the female category becomes relatively more advanced early in development than the male category (Ramsey et al., 2005). Performance in these studies requires formation of prototypes from prior experiences with multiple exemplars of faces before a preference for a prototypic (i.e., attractive) exemplar could be observed. Importantly, it appears that infants have difficulty forming prototypes of male faces and matching a series of male faces and voices together in laboratory setting; performance in prototype-formation and face-matching studies is facilitated by the use of female faces.

In addition, infants take more time to process male than female faces. These effects may stem from a relative lack of experience with male faces, given that most infants spend more time with females (e.g., their mothers), and they show perceptual preferences for female faces (Quinn et al., 2002). At the same time, male faces may be more variable in their features and in spacing between features (Hopper et al., 2014), and if the range of perceptual differences among category members is relatively wide, infant categorization is impaired (Mareschal et al., 2000). This too may make it more difficult for infants to categorize male faces. Thus differential experience and variability may systematically affect infant categorization of male and female faces. Infants may first learn to recognize and discriminate the mother’s face from other female faces, and subsequently these discrimination abilities may extend to other female faces. As a result of experience with various faces, infants should begin to form a representation of faces and a rudimentary category for faces. This representation should be most heavily weighted on the mother’s face and therefore specific to the human species, most representative of the mother’s race, and primarily female-like, so that it guides infants’ attention toward other, similar faces.

Similar effects may be operational in face race processing, which may function in accord with the contact hypothesis of social perception (Sporer, 2001). This hypothesis argues that contact with individuals from specific social groups fosters the ability to extract visual cues or invoke processing strategies that support recognition of individuals within these groups. Exposure to faces within one’s own race compared to faces of other races, for example, may lead to less practice recognizing other-race faces (cf. Johnson, 2010). Research on face categorization in infancy is consistent with this possibility. Nine-month-olds categorized faces from own- and other-races (White and Asian, respectively), yet appeared to recognize only own-race individuals (Anzures et al., 2010). This so-called “other-race effect” in recognition has been attributed to our differential experience with different categories of faces (Kelly et al., 2007).

Studies of infant and adult processing of sex and race of faces generally isolate a single social category while holding other categories constant (e.g., manipulating race while holding sex constant; e.g., Kelly et al., 2007). Research that investigates face processing when identities intersect (e.g., when a target is both Asian and male) remains relatively rare in the adult literature, and to our knowledge this issue remains largely unexplored with infants. Recent attempts to reach a more nuanced understanding of these complexities are noteworthy, and suggest that the perception of various social categories may be interdependent. For example, Quinn et al. (2008) reported that 3-month-old White infants exhibited a preference for female faces only when the faces were White, but not Asian. That is, race category may bias sex categorizations, and *vice versa*, due to common facial cues to which infants may be sensitive. (For older individuals, overlapping cognitive stereotypes may also bias race and sex categorization, but we do not expect such effects in infants.)

Such research is vital for a full account of face categorization because in the real world, people occupy multiple social categories simultaneously, and this complex intersectionality affects fundamental aspects of social perception (to be distinguished from the sociological use of the term; e.g., Browne and Misra, 2003). For example, Black men and women were judged by (predominantly White) adults as more masculine and race stereotypical than same-sex White targets. In addition, sex categorization errors, although rare, were more common for Black women than any other race/sex combination (Goff et al., 2008). There are effects of intersectionality involving emotion as well: The perceived onset and duration of happiness and anger appears to depend on both race and age. Specifically, adult observers detected anger earlier and judged it to endure longer for younger, relative to older, Black men, and observers judged happiness to disappear earlier and to be shorter lived for younger Black men. The opposite occurred for perceptions of White men (Kang and Chasteen, 2009). In addition, neutral male faces are perceived as relatively more angry than female faces, neutral White faces resemble angry expressions more than do Black or Asian faces, and neutral Black faces resemble happy expressions more than do White faces (Zebrowitz et al., 2010).

The effects of intersectionality also impact the efficiency of social categorization. Recently, Johnson et al. (2012) tested the possibility that face race will bias sex categorization through common cues and/or overlapping stereotypes, both leading to similar predictions. They theorized that a race category associated with phenotypes or stereotypes that align with the target’s sex category membership should facilitate sex categorization. This is because, for instance, Asian faces share phenotypes and/or activate stereotypes that are also common to women, and Black faces share phenotypes and/or activate stereotypes that are also common to men. A race category associated with phenotypes or stereotypes that are at odds with the target’s sex category membership, in contrast, should impair sex categorization. Specifically, male categorizations were predicted to be more efficient for Black faces, but less efficient for Asian faces, relative to White faces, and female categorizations were predicted to be less efficient for Black faces, but more efficient for Asian faces, relative to White faces. These predictions were supported in an experiment in which adult participants categorized the sex of computer-generated Asian, Black, or White faces. In a second study, the computer program employed to create these faces (FaceGen Modeler) was used to quantify the degree to which sex-typed cues covaried with race in 166 photographs of real faces. This analysis revealed that Black faces were overall more masculinized in appearance relative to Asian and White faces. Additional experiments tested implicit stereotypes held by adult observers, and confirmed that Blacks were considered to have more stereotypically male attributes (e.g., aggressive, assertive, dominant) and Asians were considered to have more stereotypically female attributes (e.g., considerate, dependent, modest). More recently, experiments have confirmed that the reciprocal relation is also true: Black categorizations were facilitated for male/masculine faces but White and Asian categorizations were facilitated for female/feminine faces (Carpinella et al., 2015). Thus sex-race intersectionality in these studies was found to operate in both a “bottom-up” (from facial characteristics) and a “top-down” (from stereotyped attributes) fashion.

The present study examines the possibility that infant face perception, likewise, is susceptible to intersectionality of sex and race. We reasoned that the tendency for infants to prefer female faces could be leveraged to examine the extent to which different face races comprise facial features that are relatively more feminine, and we tested an age range spanning important developments in race categorization to better understand how infants’ emerging sensitivity to characteristics of own- and other-races may alter such preferences. Notably, given that we test preverbal populations, the effects we report necessarily operate in the absence of stereotypes. We presented 3- and 10-month-old infants a series of face pairs in which one member of the pair was presumed to appear more feminine, and we predicted that infants would generally prefer the more feminized face. We tested this prediction in two ways. First, we manipulated facial features in FaceGen Modeler to appear explicitly female, androgynous, or male, and tested infants’ preferences for the more feminized face in pairs of Asian, Black, or White faces. Second, we manipulated face race in androgynous faces (Asian, Black, or White), and examined preferences for the face from the more “feminized” race in each pairing (Asian → White → Black). Finally, we presented blank faces (featureless ovals) which were the same average color as the androgynous faces of each race to test for inherent color preferences, to address the possibility that the hypothesized female preference might be confounded by the color of the face. Three- and 10-month-olds were chosen for observation because these age groups bracket important developments in, for example, the other-race effect (Kelly et al., 2007) and face recognition (Nelson, 2001). We reasoned that effects of intersectionality in face preference likewise might be experience-dependent, such that these effects would be stronger in the older infants. Ten-month-olds have substantially more exposure to faces than 3-month-olds, and thus more exposure to social categories and their overlapping features. We also examined differences in performance between infants from different racial groups, and effects of race of the primary caregiver, which was the mother for all infants we observed in this study.

## Materials and Methods

### Participants

Thirty-two 3-month-olds (17 boys, 15 girls; *M* = 3.2 months, SD = 0.28) and thirty-two 10-month-olds (16 boys, 16 girls; *M* = 10.0 months, SD = 0.27) composed the final sample. All infants were full term and had no known developmental difficulties. Infants were recruited from lists of birth records provided by Los Angeles County. Parents were contacted by letter and telephone, and were provided with a small thank-you gift (a toy or a T-shirt with the lab logo) for participation. An additional 34 infants were observed but excluded due to excessive fussiness or inattention (six 3-month-olds), eye-tracking calibration failures (twenty 3-month-olds, four 10-month-olds), or inability of the eye tracker to consistently track the point of gaze (one 3-month-old, three 10-month-olds).

### Materials

**FIGURE 1:**
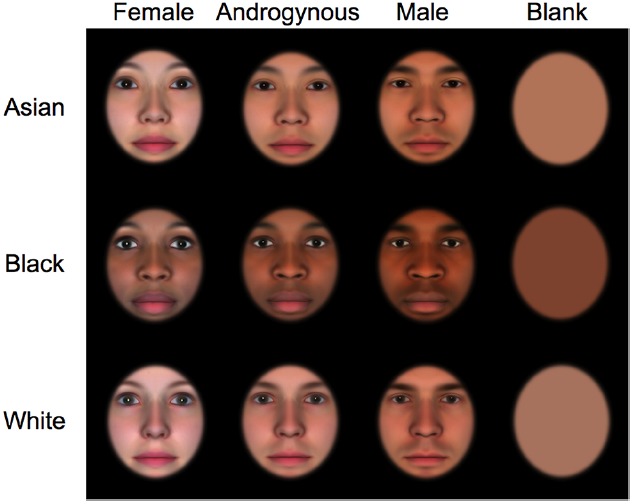
**Stimuli used in the present experiment.** Top–bottom rows: Asian, Black, and White faces. Left–right columns: female, androgynous, male, and blank faces.

A total of nine computer-generated face stimuli were created using commercial software (FaceGen Modeler) with three levels of face race (Asian, Black, or White) and three levels of face femininity (female, androgynous, or male; see Figure 1). To produce these stimuli, we first created an average androgynous White face using the Random Generation Feature of the software and by setting the gender level at the center of the 80-point femininity-masculinity scale. Using the same gender scale, we then created comparable male and female White faces, setting the scale at 60 and 20, respectively. Subsequently, Black and Asian counterparts were created by systematically manipulating the apparent race of each face using the Race Morphing Control feature of the software. Once all nine faces were generated, we used Adobe Photoshop to edit the faces. An oval-shaped outline was superimposed on each of the nine faces to expose only the internal facial features. This was necessary because we wanted to minimize the effect of external facial features on infants’ preference for a particular gender or race.

Adobe Photoshop was used to construct the final set of the stimuli, a series of side-by-side comparisons. Each visual stimulus measured 25 cm × 22.5 cm (23.5° × 21.2° visual angle) and was separated by a gap of 1.5 cm (1.4°). Each face measured approximately 14 cm × 10.5 cm (13.3° × 10.0°). For each of the three face races (i.e., White, Black, Asian), three within-race gender comparison trials were constructed (i.e., female vs. male, female vs. androgynous, and androgynous vs. male) to test infants’ preference for the more feminine faces in each comparison for all three races. In addition, three between-race comparison trials were constructed using only the androgynous faces (i.e., White vs. Black, White vs. Asian, Black vs. Asian) to examine infants’ preference for a particular face race while minimizing the potential effect of the female face preference. Furthermore, we created three additional between-race comparison trials (i.e., White vs. Black, White vs. Asian, Black vs. Asian) using blank faces (i.e., colored ovals) to examine the effect of skin tones on infants’ preference for a particular race, as noted previously. To perform such a comparison, a total of three additional blank faces were created (one per face race). The blank faces contained no facial features, and represented the average skin tone of the androgynous face.

The final stimuli of the present study consisted of two blocks of 15 side-by-side trials: nine within-race gender comparisons, three between-race comparisons using androgynous faces, and three between-race comparisons using blank faces. The left-right presentation of the faces was counterbalanced by presenting two blocks of identical trials. The second block of the trials consisted of mirror images of those in the first block. Each trial lasted for 5 s.

### Procedure

Research protocols were approved by the UCLA Institutional Review Board. Prior to testing, parents filled out a demographic questionnaire that requested information about the primary and secondary caregivers’ race. The primary caregiver for all infants in our sample was the mother. There were 33 self-identified White mothers, 8 Asians, 18 Hispanic/Latina, 2 Black, and 2 Middle Eastern. Twenty-five of the infants were categorized as White (two white parents), and 39 as mixed-race.

Each infant was observed while seated on his or her parent’s lap approximately 60 cm from a 24-inch TFT widescreen monitor (resolution set at 1900 × 1200 pixels) surrounded by black curtains to minimize distractions. Eye movements were recorded with a Tobii T60-XL eye tracker at 60 Hz with a spatial accuracy of approximately 0.5°–1°. The lights in the experimental room were dimmed and the only source of illumination came from the monitor.

Prior to their participation in the study, infants’ point of gaze was calibrated by repeated presentations of a dynamic target-patterned ball undergoing continuous contraction and expansion. The calibration stimulus was presented briefly at each of five locations on the monitor (the four corners and the center) while infants tracked it with their eyes. The Tobii eye tracker provides information about calibration quality for each point; if there were no data for one or more points or if calibration quality was poor, calibration at those points was repeated. Calibration was followed immediately by presentation of faces as described previously. Prior to each trial a small audiovisual attention-getting stimulus was shown to reorient infants’ attention to the center of the monitor.

## Results

**FIGURE 2:**
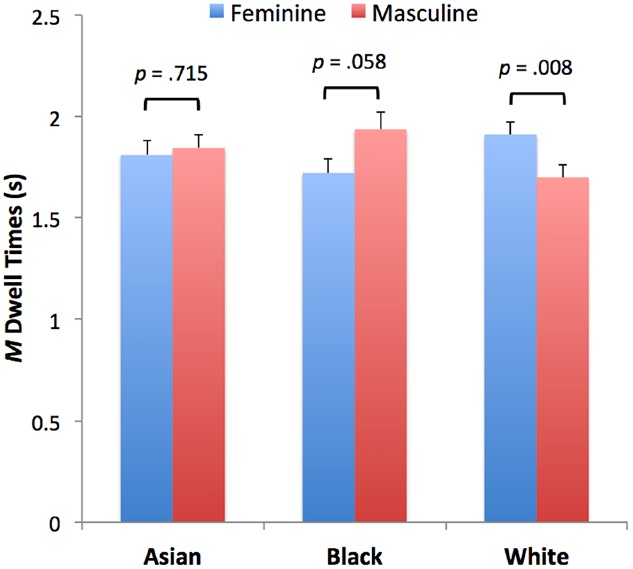
**Dwell times toward feminine vs. masculine faces in Asian, Black, and White face pairs.** Error bars = SEM.

The goal of our first set of analyses was to establish the extent to which intersectionality of sex and race characteristics influences infant preferences. We predicted greater looking toward the more feminine face in each face pairing, which we operationalized as dwell times (accumulated fixations as recorded by the eye tracker) to each of the two faces; the dependent variable for these analyses, therefore, was “femininity preference.” Our principal questions were first, whether the hypothesized female face preference would be modulated by the race of the face (i.e., Asian, Black, or White), and second, whether the female preference would be modulated by the comparisons represented by each pairing (i.e., female–male pairings, female–androgynous pairing, and androgynous–male pairings). We also examined age differences in performance to assess the possibility that infants’ preferences and potential intersectionality effects may emerge in parallel with other key face processing skills (Nelson, 2001; Kelly et al., 2007). We computed a 3 (Comparison: female–male, female–androgynous, or androgynous–male) × 3 (Face Race: Asian, Black, or White) × 2 (Age Group) × 2 (“Femininity” Preference in each pairing) mixed ANOVA with repeated measures on the last factor. This analysis yielded a statistically significant main effect of Comparison, *F*(2,124) = 3.20, *p* = 0.044, ${\text{η}}_{p}^{2}$ = 0.049, stemming from longer overall looking at female–male than at androgynous–male pairings and somewhat more at female–androgynous than at androgynous–male pairings (the reasons for these effects are unclear). More importantly, we found a significant Face Race × Femininity Preference interaction, *F*(2,124) = 5.06, *p* = 0.008, ${\text{η}}_{p}^{2}$ = 0.075 (see Figure 2). Tests for simple effects revealed that infants looked longer at the more feminine face when faces were White, *F*(1,63) = 7.61, *p* = 0.008, but not when faces were Asian, *F*(1,63) = 0.13, *p* = 0.715. When faces were Black, in contrast, there was a trend toward a *male* preference, *F*(1,63) = 3.72, *p* = 0.058. Additional simple effects tests revealed that the preference for feminine faces was reliably stronger for White vs. Black faces, *F*(1,62) = 8.80, *p* < 0.01, and marginally stronger for White vs. Asian faces, *F*(1,62) = 3.47, *p* = 0.067. There were no other significant main effects or interactions. These data, therefore, demonstrate that the female face preference reported in earlier studies (e.g., Quinn et al., 2002) is contingent on face race, having been observed under tested conditions only when infants viewed White faces (cf. Quinn et al., 2008).

The next analysis examined the possibility that infants perceive Asian, Black, and White race faces to be gendered, as has been reported for different race face morphologies in photographs of real faces (Johnson et al., 2012). If so, we predicted that, when gender cues have been equated except for face race (i.e., in androgynous faces), faces that are relatively more feminized would be preferred by virtue of the hypothesized intersectionality of face race and gender. Specifically, we predicted that Asian androgynous faces will be preferred to White and Black, and White androgynous faces will be preferred to Black because faces may be gendered by race rather than by direct manipulation of sex-typed facial features within FaceGen Modeler. A 3 (Comparison: Asian vs. White, Black vs. Asian, Black vs. White) × 2 (Femininity Preference in each pairing) × 2 (Age Group) mixed ANOVA revealed a statistically significant main effect of Age Group, *F*(1,62) = 6.27, *p* = 0.015, ${\text{η}}_{p}^{2}$ = 0.092, due to overall longer dwell times by 10-month-olds (*M* = 11.64 s, SD = 2.51) vs. 3-month-olds (*M* = 10.18 s, SD = 2.15). There were no other significant main effects or interactions. These analyses provide evidence against the likelihood that different race faces appear differently gendered to infant observers.

**FIGURE 3:**
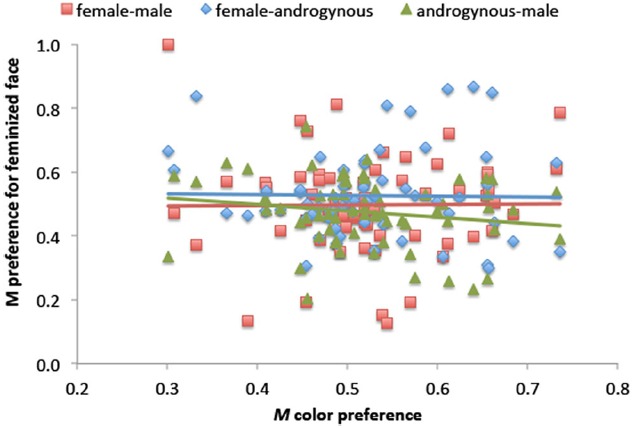
**Correlations between ***M*** color preference (i.e., preference for darker colors) and preference for feminized faces in comparisons of female vs. male (red), female vs. androgynous (blue), and androgynous vs. male (green).** Corresponding colored trend lines are superimposed.

A third set of analyses examined the possibility that differences in skin color between Asian, Black, and White faces may have influenced infants’ preferences. To achieve this goal, we examined preferences for the darker blank face in Asian–White, Black–Asian, and Black–White pairings with a 3 (Comparison) × 2 (Skin Tone Preference: darker vs. lighter face) × 2 (Age Group) mixed ANOVA, which revealed a statistically significant main effect of Skin Tone Preference, *F*(1,62) = 4.66, *p* = 0.035, ${\text{η}}_{p}^{2}$ = 0.070, the result of longer looking overall at darker faces (*M* = 1.55 s, SD = 0.50) relative to lighter faces (*M* = 1.42 s, SD = 0.40), and a main effect of Age Group, *F*(1,62) = 8.55, *p* = 0.005, ${\text{η}}_{p}^{2}$ = 0.121, due to overall longer dwell times by 10-month-olds (*M* = 9.81 s, SD = 2.26) vs. 3-month-olds (*M* = 8.02 s, SD = 2.63). There was also a reliable three-way interaction, *F*(2,124) = 6.45, *p* = 0.002, ${\text{η}}_{p}^{2}$ = 0.094, stemming from somewhat stronger dark preferences by 3-month-olds viewing the Black–Asian comparison and by 10-month-olds viewing the Black–White comparison. In addition, we used correlation analyses to examine relations between the *M* color preference for individual infants and femininity preferences in face pairs (female–male, female–androgynous, and androgynous–male) in light of our previously described results showing that infants’ female face preference is modulated by face race. These analyses revealed no statistically reliable effects, *p*s > 0.172 (see Figure 3). Taken together, these analyses reveal that infants tended to prefer darker colors, but this preference did not interact with face race, and there was no consistent way in which color preference *per se* was related to female preference.

Finally, we examined differences in face preference as a function of the mother’s and the infant’s race with a series of Bonferroni-corrected *t*-tests. There were no statistically significant differences in any of the preferences we reported previously in this section between infants of White mothers (*n* = 33) vs. infants of Asian, Hispanic, Black, or Middle Eastern mothers (*n* = 31), nor were there any reliable differences in preference between infants from White (*n* = 25) vs. mixed-race (*n* = 39) families.

## Discussion

We examined the hypothesis that facial features specifying race and gender may overlap to the extent that infants perceive faces to be gendered (as do adults; Johnson et al., 2012) by capitalizing on the previously-reported tendency of infants to prefer female faces (Quinn et al., 2002, 2008). We tested infants’ visual preferences for female vs. male in Asian, Black, and White computer-generated face pairs, we tested preferences for Asian vs. White, Asian vs. Black, and White vs. Black in pairs of androgynous faces, and we tested for preferences for oval patches of color that represented the average hue of Asian, Black, and White androgynous faces. Specifically, we tested the possibilities that (a) infants’ purported female face preference would vary as a function of face race, and (b) that race faces are inherently gendered due to phenotypic overlap in facial features that are characteristic of sex differences. The first hypothesis, but not the second, was supported, and we interpret these two findings in turn.

Consider first the results of analyses of the female preference in different race face pairs. The female preference we predicted was observed in White face pairs, but not in Asian or Black face pairs. This result replicates and extends findings of Quinn et al. (2008), who discovered that White 3-month-olds preferred female faces only when the faces were White, but not Asian. Here, we found the same result across the sample, even among infants who were not White or who came from mixed-race families. Interestingly, we found also that the female preference is actually reversed to an extent when infants view Black faces. We showed also that 10-month-olds’ visual preferences were not statistically different from those of 3-month-olds. This result implies that developments in the other-race effect (Kelly et al., 2007), which likely stem from growing experience with same-race faces during the first year after birth (cf. Scott et al., 2007) had little bearing on infants’ behavior under tested circumstances; nor did races of household members seem to matter, in contrast to the findings reported by Quinn et al. (2002).

It may be that infants did not exhibit the female preference in Asian face pairs because sexual dimorphism in Asian faces is reduced relative to White faces—that is, the differences between female and male facial features is greater in Whites. Hopper et al. (2014) used multidimensional (MDS) scaling to place 40 photographs of Asian and White women and men (10 photos each) into a “face space,” so that different facial attributes (dimensions of facial features and distances between features) corresponded to distinct dimensions within the MDS scaling space. Gender was found to vary more for White faces, resulting in a negative or positive correlation between gender and race when only considering male or only considering female faces. Female and male Asian faces, therefore, are relatively more similar in appearance, and this may mean it is somewhat less likely that infants can discriminate female from male, or that they are not sufficiently distinct in appearance such that females attract more attention. It may be that infants did not exhibit the female preference in Black face pairs because of superficial similarities between the characteristics of neutral male and neutral Black faces to happy expressions in general, as revealed by outputs of connectionist models trained to recognize facial metrics of angry, happy, and surprise expressions in White male and female faces (Zebrowitz et al., 2010). If phenotypic characteristics of Black faces (in particular, Black male faces) overlap with positive expressions, which are known to attract infants’ attention relative to other emotions (e.g., Kim and Johnson, 2013, 2014), then a reduction in female preferences in Black faces (indeed, nearly to the point of statistical significance in the other direction) may stem from a latent tendency for Black faces to convey positive emotions, even though the computer-generated faces used in the present experiment were explicitly neutral with respect to emotional expression. It remains for future research to examine more carefully the possibility of intersectionality of race and emotion in infant face perception.

Consider next our second principal question in the present study, the possibility that face race is inherently gendered, again due to purported overlap in facial features that convey information for attributes specifying race and sex. We found no evidence under tested circumstances that infants perceived Asian and White faces to be relatively more feminized, or Black faces to be relatively more masculinized, as has been reported from experiments with adult observers and from detailed measurements of facial features in photographs (Johnson et al., 2012). We observed no age differences between 3- and 10-month-olds in infant female preferences, nor did we observe differences in visual preferences as a function of infant race (White or non-White). It may be that the conditions we employed to test this question involved distinctions in face race that were too fine-grained to be detected by infants in androgynous faces, or it may be that this kind of race-gender overlap awaits developments in perceptual skills that occur beyond infancy. Notably, adult responses to race-gender intersectionality are highly sensitive to both bottom-up (feature overlap) and top-down (stereotypicality) effects, as observed with reaction time, mouse tracking, and verbal judgments (Johnson et al., 2012). Given the importance of the top-down effects that Johnson et al. (2012) reported, however, it is possible that sensitivity to some subtle facial cues supporting race and gender distinctions emerge in tandem with the cognitive representations that underlie stereotypes, in-group preferences, and racial and gender biases (cf. Hugenberg et al., 2010; Young et al., 2012). For example, it has been proposed that attributes that distinguish among social groups attain “psychological salience” in childhood (Bigler and Liben, 2007), and this may tune the visual system toward certain physical characteristics that then become perceptually salient (cf. Scott et al., 2007).

### Conflict of Interest Statement

The authors declare that the research was conducted in the absence of any commercial or financial relationships that could be construed as a potential conflict of interest.
